# Roles of peripheral lipoproteins and cholesteryl ester transfer protein in the vascular contributions to cognitive impairment and dementia

**DOI:** 10.1186/s13024-023-00671-y

**Published:** 2023-11-16

**Authors:** Tetiana Poliakova, Cheryl L. Wellington

**Affiliations:** 1 Department of Pathology and Laboratory Medicine, 2215 Wesbrook Mall, Vancouver, BC V6T 1Z3 Canada; 2 Djavad Mowafagian Centre for Brain Health, University of British Columbia, Vancouver, BC Canada; 3 International Collaboration On Repair Discoveries, Vancouver, BC Canada; 4 School of Biomedical Engineering, University of British Columbia, Vancouver, BC Canada

**Keywords:** Alzheimer’s Disease, Vascular contributions to cognitive impairment and dementia, Low density lipoprotein, High density lipoprotein, Cholesteryl ester transfer protein

## Abstract

This narrative review focuses on the role of cholesteryl ester transfer protein (CETP) and peripheral lipoproteins in the vascular contributions to cognitive impairment and dementia (VCID). Humans have a peripheral lipoprotein profile where low-density lipoproteins (LDL) represent the dominant lipoprotein fraction and high-density lipoproteins (HDL) represent a minor lipoprotein fraction. Elevated LDL-cholesterol (LDL-C) levels are well-established to cause cardiovascular disease and several LDL-C-lowering therapies are clinically available to manage this vascular risk factor. The efficacy of LDL-C-lowering therapies to reduce risk of all-cause dementia and AD is now important to address as recent studies demonstrate a role for LDL in Alzheimer’s Disease (AD) as well as in all-cause dementia. The LDL:HDL ratio in humans is set mainly by CETP activity, which exchanges cholesteryl esters for triglycerides across lipoprotein fractions to raise LDL and lower HDL as CETP activity increases. Genetic and pharmacological studies support the hypothesis that CETP inhibition reduces cardiovascular risk by lowering LDL, which, by extension, may also lower VCID. Unlike humans, wild-type mice do not express catalytically active CETP and have HDL as their major lipoprotein fraction. As HDL has potent beneficial effects on endothelial cells, the naturally high HDL levels in mice protect them from vascular disorders, likely including VCID. Genetic restoration of CETP expression in mice to generate a more human-like lipid profile may increase the relevance of murine models for VCID studies. The therapeutic potential of existing and emerging LDL-lowering therapies for VCID will be discussed.

**Graphical Abstract:**

Figure Legend*.* Cholesteryl Ester Transfer Protein in Alzheimer’s Disease. CETP is mainly produced by the liver, and exchanges cholesteryl esters for triglycerides across lipoprotein fractions to raise circulating LDL and lower HDL as CETP activity increases. Low CETP activity is associated with better cardiovascular health, due to decreased LDL and increased HDL, which may also improve brain health. Although most peripheral lipoproteins cannot enter the brain parenchyma due to the BBB, it is increasingly appreciated that direct access to the vascular endothelium may enable peripheral lipoproteins to have indirect effects on brain health. Thus, lipoproteins may affect the cerebrovasculature from both sides of the BBB. Recent studies show an association between elevated plasma LDL, a well-known cardiovascular risk factor, and a higher risk of AD, and considerable evidence suggests that high HDL levels are associated with reduced CAA and lower neuroinflammation. Considering the potential detrimental role of LDL in AD and the importance of HDL’s beneficial effects on endothelial cells, high CETP activity may lead to compromised BBB integrity, increased CAA deposits and greater neuroinflammation.

Abbreviations: CETP – cholesteryl transfer ester protein; LDL – low-density lipoproteins; HDL – high-density lipoproteins; BBB – blood-brain barrier; CAA – cerebral amyloid angiopathy, SMC – smooth muscle cells, PVM – perivascular macrophages, RBC – red blood cells.

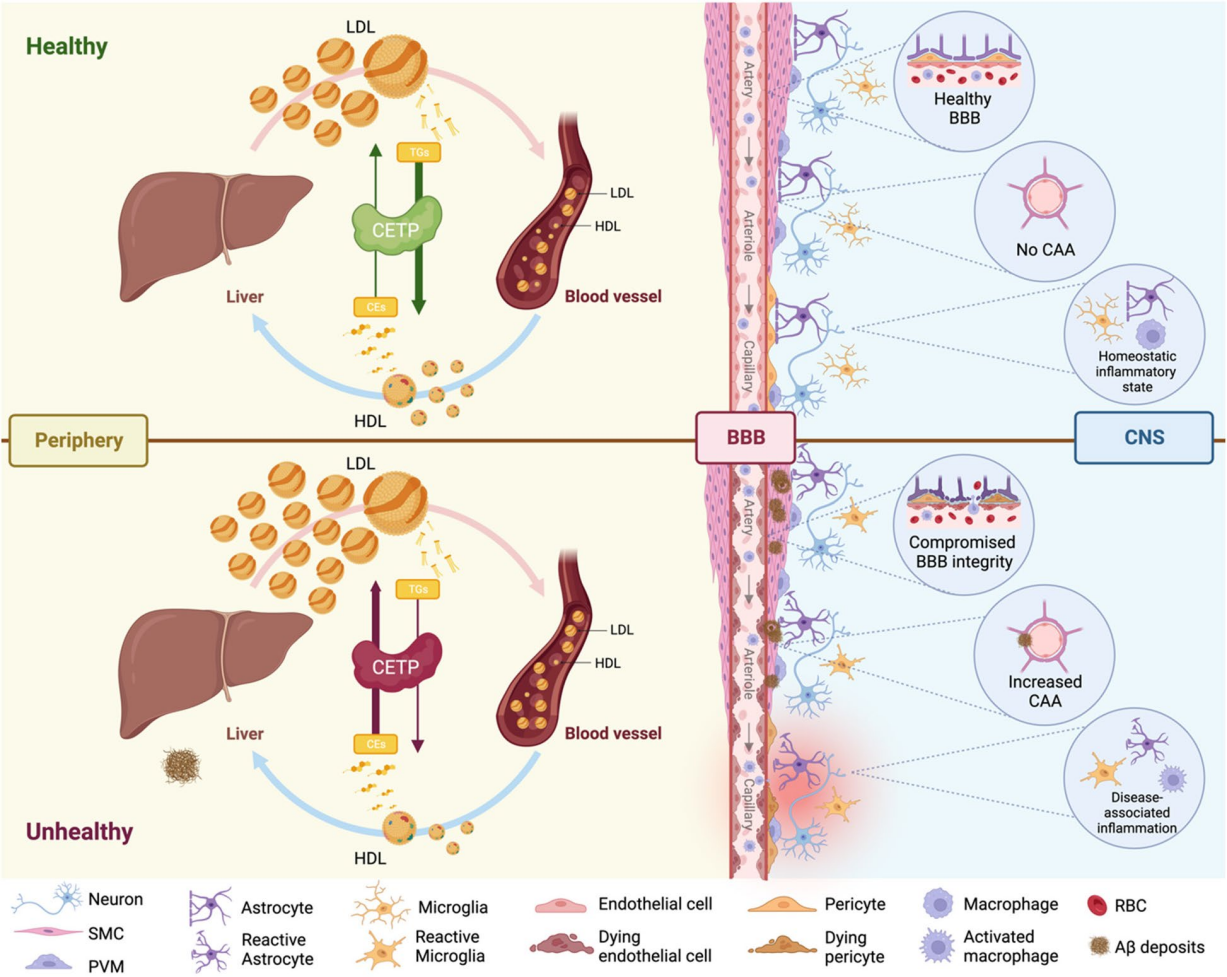

## Background

Cardiovascular risk factors play an undeniable role in the vascular contributions to cognitive impairment and dementia (VCID) [1], particularly for Alzheimer’s Disease (AD), the most common type of dementia, where cerebral vessels have key roles in clearing amyloid beta (Aβ) peptides from the brain [2, 3]. The issues under discussion for this narrative review include: 1) the association of major peripheral lipoprotein subclasses, focusing on low-density lipoprotein (LDL) and high-density lipoprotein (HDL), on VCID and AD; 2) current challenges in using murine models to investigate mechanisms by which LDL and HDL contribute to VCID; and 3) describing the functions of cholesteryl ester transfer protein (CETP) in regulating the LDL:HDL ratio in humans, including therapeutic strategies targeting CETP. The literature search strategy used focused on the search terms AD, VCID, LDL, HDL, and CETP to identify primary papers and review articles in PubMed and Medline within the last 25 years. As this is a narrative review, formal inclusion and exclusion criteria to guide selection of articles for in-depth review were not used. Nevertheless, both authors reached consensus on the articles reviewed to ensure a balanced representation of primary findings.

### Alzheimer’s Disease and the cerebrovasculature

Alzheimer’s Disease (AD), the leading cause of dementia, is definitively diagnosed upon neuropathological evidence of amyloid plaques consisting of fibrillar beta-amyloid (Aβ) peptides that deposit in the brain parenchyma and cerebral vessels, as well as neurofibrillary tangles consisting of aggregated hyperphosphorylated tau protein that deposits within neurons [4]. As neurological health depends on a properly functioning neurovascular unit, it is not surprising that there is a close relationship between vascular health and AD. Despite comprising only 2% of total body mass, the brain consumes approximately 20% of total cardiac output and contains over 400 miles of blood vessels [5]. Age, sex, smoking, blood pressure, physical activity, type 2 diabetes mellitus and dyslipidemia are well-established AD risk factors [6–8]. Favorable cardiovascular health and management of modifiable vascular risk factors, especially at midlife, attenuates dementia risk decades later [9–11].

Large autopsy studies show that cerebrovascular pathologies are common in AD [12]. Specifically, 60 to 90% of AD brains have evidence of cerebral vessel disease, including arteriole and precapillary deformities [13], reduced vascular density [14, 15], increased vessel tortuosity [15], vessel remnants that lack endothelial cells [16–18], and accumulation of Aβ in cerebrovascular arteries, arterioles and capillaries, known as cerebral amyloid angiopathy (CAA) [19]. Studies by the National Alzheimer’s Coordinating Center and the Religious Orders Study and Rush Memory and Aging Project found a greater burden of macro- and micro-infarcts, atherosclerosis, arteriosclerosis and CAA in AD compared to other neurodegenerative diseases [20], and increased AD risk in cases with infarcts and more severe atherosclerosis or arteriosclerosis [21], respectively. CAA is also present in up to 40% of cognitively healthy elderly brains [22]. CAA may reflect impaired clearance of Aβ, which is removed from the brain through multiple pathways including active transport across brain endothelial cells in a process involving LDL receptor related protein (LRP1) [23], p-glycoprotein [24] and LDL receptor [25], and by perivascular drainage in mid- and large-sized arteries along smooth muscle cell basement membranes [26, 27].

Together, this body of literature suggests that strategies that reduce cardiovascular risk and promote cerebrovascular resilience could potentially also reduce AD risk, particularly for patients who have vascular comorbidities. AD has a long prodromal period where neuropathological changes begin to occur 15 to 20 years prior to clinical onset of memory problems, which typically emerge in the 6^th^ and 7^th^ decade of life [28]. This provides a relatively large window of opportunity for interventions that target vascular factors to be evaluated as primary or secondary prevention strategies to delay progression to clinical dementia.

There is tremendous interest in this topic given that several anti-amyloid immunotherapies have been approved by the Food and Drug Administration (FDA) for mild AD [29–32]. Although these drugs promote clearance of amyloid from the brain largely via perivascular pathways and can slow cognitive decline, they are also associated with safety concerns known as amyloid related imaging abnormalities (ARIA) including edema (ARIA-E) and hemorrhage (ARIA-H) subtypes that reflect damage to cerebral vessels during amyloid clearance [33, 34]. ARIA-E can also arise spontaneously as an autoimmune encephalopathy in patients with CAA who have vascular or perivascular inflammatory infiltrates [35, 36] and CSF anti-Aβ autoantibodies [37]. Increased efforts to screen large cohorts for anti-amyloid clinical trials are beginning to identify rare cases or ARIA-E that appear to be more common in patients with CAA or who carry genetic risk factors for high vascular amyloid burden, such as the presence of apoE4 alleles [38]. It will be important to learn whether improving vascular risk factors, including the LDL:HDL ratio, prior to or concurrent with anti-amyloid immunotherapies, also may improve safety and efficacy.

### Lipoproteins in AD

*Apolipoprotein E (ApoE):* ApoE is the most significant genetic risk factor for sporadic late onset AD, with apoE2 (Cys112, Cys158) protective, apoE3 (Cys112, Arg158) neutral, and apoE4 (Arg112, Arg158) detrimental [39]. The mechanisms by which apoE affects AD risk and age of onset remain incompletely understood. Although apoE4 is most well-known for accelerating amyloid deposition [40, 41], apoE also regulates tau-mediated neurogeneration [42] and has pleiotropic effects on cerebral vessels including regulating BBB integrity [43], amyloid clearance across the BBB [41], vascular transcriptomic and proteomic signatures [44], cerebral blood flow [45], inflammation [46, 47], vascular compliance via modifying endothelial nitric oxide secretion [48, 49], and brain glucose metabolism [50].

In addition to apoE produced within the central nervous system (CNS), apoE is also made by peripheral hepatocytes and macrophages and, as it is an exchangeable lipoprotein, circulates in blood on several peripheral lipoprotein subclasses [51]. Notably, peripheral lipoproteins have largely been ignored in AD as the healthy BBB prevents brain and peripheral apoE pools from mixing [52, 53]. However, it is increasingly appreciated that peripheral lipoproteins, including those containing apoE, can affect brain function via their actions on the cerebrovasculature [54–56]. Importantly, the underlying mechanisms by which lipoproteins on either side of the BBB affect cerebrovascular functions, including regulation of cerebrovascular reactivity and amyloid clearance pathways, are largely unknown. It is reasonable to hypothesize that peripheral lipoproteins may be important in regulating endothelial functions as their circulation through the vascular lumen allows direct endothelial contact.

Both the quality and quantity of peripheral apoE may matter in AD pathogenesis. In humans, patients with prodromal and manifest AD have lower plasma apoE levels compared to cognitively healthy controls, and the *APOE4* genotype is associated with lower plasma apoE levels regardless of the diagnosis [57]. Moreover, low plasma apoE levels are associated with Aβ pathology, higher cerebrospinal fluid (CSF) total- and phosphorylated-tau and worse cognition [57]. Mice engineered to have inducible liver-specific human apoE3 and apoE4 expression with no detectable apoE in the brain further support the protective effects of peripheral apoE3 and the toxic effects of peripheral apoE4 on brain function [58]. Transcriptomic analysis reveals that peripheral expression of apoE4 leads to endothelial dysfunction and impaired immune function within the glio-vascular unit that disrupts vascular function and BBB integrity [58]. In APP/PS1 mice, peripheral expression of apoE3 reduces brain amyloid pathology while apoE4 exacerbates it [58]. Thus, lipoproteins may affect the cerebrovasculature from either side of the BBB; brain lipoproteins made largely from apoE acting from the abluminal side and peripheral lipoproteins acting from the luminal side. This concept highlights the cerebrovasculature as an important functional bridge between the brain and the rest of the body.

*Low-density lipoprotein (LDL):* LDL particles exist in subclasses including large, buoyant LDL-I (density 1.019–1.023 g/mL), LDL-II of intermediate size and density (density 1.023–1.034 g/mL), small dense LDL-III (density 1.034–1.044 g/mL), and a fourth subfraction of very small dense LDL-IV (density 1.044–1.063 g/mL), which is present in individuals with elevated triglyceride (TG) levels [59–62]. In humans, it is well established that elevated levels of LDL cholesterol (LDL-C) are causally related to risk of cardiovascular disease including atherosclerosis, coronary artery disease, stroke, hypertension and type II diabetes [63] and that lowering levels of LDL and other apoB-containing lipoproteins reduces cardiovascular events [64]. In large peripheral vessels, LDL can cross the endothelium and enter the arterial intima [65] via electrostatic interactions with arterial proteoglycans [66], however, whether this process occurs in leptomeningeal or other cerebral vessels is not known. Importantly, oxidized LDL damages the endothelial barrier integrity [67, 68], which may be particularly relevant to the cerebral microvasculature. With respect to the impact of biological sex on AD risk, estrogens reduce LDL transcytosis by downregulating scavenger receptor B1 (SR-B1) in endothelial cells [69] and, if this process also occurs in cerebral vessels, may account for some of the increased risk for AD in post-menopausal women.

A recent meta-meta analysis of plasma lipids in AD supports a significant association between high LDL-C levels with increased AD risk [70]. A UK Biobank study of more than 1.8 million people over two decades revealed a modest association between dementia risk and LDL-C measured in mid-life (< 65 years) [71]. A meta-analysis and systematic review that included this and two additional cohort studies investigating the association between midlife LDL and HDL levels and dementia concluded that for each 1 mmol/L increase in LDL-C, there is an 8% increase in incidence of all-cause dementia [72]. The Religious Orders Study/Memory and Ageing Project further demonstrated that LDL-C is associated with AD neuropathology including Aβ plaques and neurofibrillary tangles and CAA independent of apoE. Therefore, LDL-C is a modifiable risk factor for AD.

*High density lipoproteins (HDL):* HDL is a peripheral lipoprotein class that regulates reverse cholesterol transport, a process that removes excess cholesterol from the body [73]. The human HDL “particle-ome” contains ~ 200 proteins, ~ 300 lipids, and RNA cargo [74, 75]. HDL composition differs among individuals and disease states [76–79] and becomes less cardioprotective in women after menopause [80]. Although clinical measures of HDL’s cholesterol content, HDL-C, remains a gold standard to quantify HDL *levels*, HDL *functions* are determined by their composition [81], with different proteins driving distinct functions of HDL subpopulations [82]. Circulating HDL has multiple vasoprotective functions including stimulating reverse cholesterol transport, reducing endothelial activation, and mitigating endothelial inflammation [76, 83]. While HDL has been extensively studied in cardiovascular disease [84, 85], its potential roles in AD are less clear [76, 83]. High levels of HDL-cholesterol (HDL-C) or apoA-I (HDL’s major protein) correlate with reduced AD risk [86], improved memory [87, 88] and low brain amyloid [89]. The prospective Honolulu-Aging study found that the highest quartile of plasma apoA-I at baseline correlated with the lowest risk of dementia 16 years later [90]. Similarly, high baseline HDL-C in the Baltimore Longitudinal Study of Aging protected against cognitive impairment and brain volume reductions 20 years later [91]. However, cross-sectional studies including the Framingham Heart Study [92] and others [93], and some prospective studies with short follow-up [94–97], found no relationship between HDL-C and cognitive impairment. It is likely that baseline age and follow-up length may explain these inconsistencies [90, 95], as studies with > 10 years of follow-up found significant associations between HDL-C levels and AD risk [90, 91], whereas those with < 10 years of follow-up did not [95, 97]. Furthermore, baseline HDL-C levels in middle age were significantly associated with AD risk [87, 90, 98], whereas baseline measures in subjects > 70 years were not [96, 97]. Thus, HDL, like LDL, may exert its greatest influence on AD risk at mid-life. New findings report a significant interaction between low serum HDL-C and cognitive impairment in *APOE4* carriers [99], which contrasts with another study that found an association between genetically determined increased HDL-C and higher risk of AD using a Mendelian randomization framework [100]. Contradictory conclusions of HDL’s role in AD pathogenesis may be prompted by the composition and functionality of HDL particles and not simply by HDL-C levels in plasma, as well as when in life HDL is measured.

The importance of understanding the functional properties of plasma HDL subpopulations has gained considerable attention in recent years. ApoE is present in a minor subfraction (6–9%) of total circulating HDL particles and was previously believed to mainly recycle these HDL particles in the liver [101, 102]. However, the fraction of HDL that contains apoE (HDL-apoE) is now believed to promote critical metabolic steps in reverse cholesterol transport that are associated with reduced coronary heart disease risk [82], and plasma HDL-apoE was recently confirmed as a potential biomarker for coronary heart disease [103]. Recently, high HDL-apoE levels were found to be associated with improved cognitive function as measured by the Modified Mini-Mental State Examination in the elderly (> 75 years of age) [104], and high levels of plasma HDL-apoE lacking apoC-III were reported to be associated with better cognitive function and lower dementia risk in a prospective case-cohort of 1351 participants in the Ginkgo Evaluation of Memory Study [105]. HDL-apoE can also reduce vascular stiffening by altering expression of the extracellular matrix proteins collagen-I and fibronectin in vascular smooth muscle cells [106].

Preclinical studies in mice show that deletion of apoA-I in amyloidogenic models drastically reduces HDL levels and exacerbates CAA and cerebrovascular inflammation [33, 38]. Conversely, apoA-I overexpression from its native promoter reduces CAA and neuroinflammation [20], and delivery of recombinant HDL or apoA-I Milano (an atheroprotective apoA-I genetic variant) into the systemic circulation of mice acutely decreases soluble brain Aβ levels and leads to long-lasting lowering of CAA and neuroinflammation, respectively [13, 14], suggesting the potential for HDL to play a role in removing pre-existing vascular amyloid deposits in vivo.

In a scaffold-directed model of perfusable cerebral blood vessels, HDL delivered from the “blood side” facilitates Aβ transport and attenuates Aβ accumulation in the synthetic vascular tissue, particularly for more pathogenic Aβ42 peptide [107], and the HDL-apoE fraction appears particularly potent for this function [108]. Multiple mechanisms appear to underlie HDL’s ability to attenuate Aβ vascular deposition and Aβ-induced endothelial inflammation, namely reducing Aβ binding to collagen-I by forming an HDL-Aβ complex, reducing collagen-I protein levels produced by smooth-muscle cells (SMC), and blocking Aβ uptake into SMC perhaps by reducing LRP1 levels [108]. In this model system, HDL appeared ineffective in reducing preformed vascular Aβ deposits in a 24 h time period [109], suggesting that either more time may be required to attenuate pre-existing CAA or that components in addition to HDL may be required in this model system, including pericytes that have a clear role in CAA pathology [110].

### Sex differences in LDL and HDL

It is well-established that two thirds of those with AD are women and that menopause is associated with a shift from a cardioprotective lipid profile to an atherogenic lipid profile characterized by higher LDL-C and lower and more dysfunctional HDL-C in post-menopausal women [111–114]. A recent study of 5,366 statin users from approximately 50,000 participants in the real-world PharmLines initiative in the Netherlands showed that women had a significantly higher mean percent increase in HDL-C levels upon statin therapy compared to men, with no sex differences observed for LDL-C reduction [115]. However, another well-powered primary care study found that women were less adherent to statin treatment compared to men [116]. Sex but not menopausal differences were also observed in a small real-world study of proprotein convertase subtilisin/kexin type 9 (PCSK9) inhibitors, another class of LDL-C-lowering therapies [117]. Much remains to be learned about how targeting vascular risk factors for primary and secondary prevention of dementia may require sex and menopausal considerations.

### Cholesteryl ester transfer protein (CETP)

CETP is a boomerang-shaped glycoprotein produced primarily by the liver [118, 119] that promotes bidirectional transfer of hydrophobic lipids, cholesteryl esters and triglycerides across plasma lipoprotein subclasses [120]. Because most of the cholesteryl esters in plasma are found in HDL and most triglycerides originate from very low density lipoprotein (VLDL) and chylomicrons, the net effect of CETP activity is a net mass transfer of cholesteryl esters from HDL to pro-atherogenic LDL and VLDL particles, and a net mass transfer of triglycerides from triglyceride-rich VLDLs and chylomicrons into HDL and LDL [121]. CETP is primarily expressed in and secreted from in the liver and is catalytically active in humans, non-human primates, rabbits and hamsters, but is absent in many other species [122]. In humans, brain CETP expression is very low in most regions, as the Human Protein Atlas human brain dataset lists less than 2 normalized transcript per million (nTPM), the GTEx human brain RNA-Seq dataset shows < 1 nTPM in all brain regions except for the pituitary, and the FANTOM5 human brain CAGE dataset shows ~ 18 scaled tags per million in retina but less than 2 in brain regions [123, 124]. This lack of clear expression in the human brain suggests that CETP may exert its CNS effects indirectly, likely via the cerebrovasculature.

#### CETP polymorphisms

According to the genome Aggregation Database (gnomAD), hundreds of single nucleotide variants (SNVs) in the *CETP* gene have been identified, including 125 synonymous SNVs, 290 missense SNVs and 33 predicted loss-of-function variants [125]. Because of CETP’s ability to modulate lipid transport, the natural genetic variations at the *CETP* locus on CETP activity, protein and lipid levels, and impact on disease have been widely studied. Data from the UK Biobank and the CARDIoGRAM plus C4D consortium show that the CETP genetic score comprising of *CETP* variants associated with higher HDL (rs3764261, rs1800775, rs708272, rs9939224, rs2303790) have biologically equivalent effects to LDL-C-lowering therapies on reducing the risk of major coronary events when measured per unit change in apoB [126]. In another study aimed to identify regulatory elements affecting CETP mRNA expression, allelic mRNA expression in 56 human livers was shown to be strongly associated with three upstream promoter/enhancer SNPs (rs173539, rs247616, rs3764261) [127]. A closer examination of molecular mechanisms revealed the minor allele of rs247615 and associated high linkage SNPs lead to reduced expression across tissues, potentially because rs247616 alters the putative binding sites of highly expressed transcription factors: Y-box binding protein 1 (YBX1) and CCAAT/enhancer binding protein alpha (CEBPA) [128].

The same group also investigated the common exon 9-lacking alternative splicing isoform ($\Delta$ 9), which prevents CETP secretion in a dominant negative manner. The $\Delta$ 9 splice variant was found within 10–48% of total CETP mRNA in 94 livers analysed and was strongly associated with rs5883 and rs9930761 in complete linkage disequilibrium. In males, both rs247616 and rs5883T/rs9930761C were independently associated with elevated HDL-C levels with similar effect sizes. Notably, males with rs5883T/rs9930761C also had significantly increased risk for myocardial infarction (MI), stroke and all-cause mortality. Neither polymorphism had a significant effect in females. This suggests that low CETP activity is associated with poor cardiovascular disease (CVD) outcomes in males and that there are sex-dependent CETP splicing effects independent of HDL levels [127].

A recent study investigating the effect of five single-nucleotide polymorphisms (SNPs; rs1532624, rs5882, rs708272, rs7499892, and rs9989419) and haplotypes on the cardiovascular risk (CVR) in 368 Hungarian/Roma samples showed a significant association between the T allele of rs7499892 and increased CVR estimated by the Framingham Risk Score. Furthermore, three out of 10 haplotypes (H5, H7, and H8) investigated also showed a significant correlation with increased CVR. Interestingly, the H5 effect was mediated via TG and HDL-C levels, while the impact of H7 and H8 was independent of TG and HDL-C, suggesting that more mechanisms need to be explored [129]. Rs708272, which showed no significant association with CVR in the study described above, also failed to show an association with MI risk in 2286 patients from Western Siberia [130] which is inconsistent with an earlier meta-analysis concluding that rs708272 may contribute to MI susceptibility among Caucasians but not Asians [131]. Interestingly, a meta-analysis by Thompson et al. suggests the opposite, a weakly inverse association with coronary risk for rs708272 and two more CETP-inhibiting SNPs (rs5882 and rs1800775) [132].

The discrepancies between the effects of rs708272 and many other *CETP* polymorphisms (Table 1) can be explained by several challenges. Firstly, the overwhelming majority of genetic studies investigating *CETP* variants are candidate gene analyses which have been criticized for low statistical power, incomplete coverage of relevant genetic variation within candidate genes and potentially confounding influences such as environmental and ethnic backgrounds [133]. Secondly, CETP’s complex biology may serve as a limitation to genetic association interpretations. Boekholdt and Thompson point out that although CETP activity is often measured to determine the effect of a given polymorphism, protein levels in tissues may contribute to disease outcomes as well. CETP mass measurements could also potentially serve as a limitation if antibodies have different affinities to protein variants [134]. Lastly, most CVDs represent a complex interplay between lifestyle and environmental risk factors with many contributing genes with multiple polymorphisms, suggesting that a given *CETP* polymorphism may have a strong effect in one population but not necessarily in a different population.

**Table 1:** Common CETP single-nucleotide polymorphisms and their impact on cardiovascular risk and Alzheimer’s Disease

CETP SNPs	Effect on CETP activity	Effect on cardiovascular risk	Effect on AD risk
rs247616 (C > T)	Unknown, but decreased CETP mRNA expression [127] and increased HDL [170]	Decreased CAD risk [171]	No association [172]
rs1800775 (-629C > A)	Decreased [132]	Mixed: Increased MI risk [131] Higher CAD risk [173] Reduced IHD, MI, ICVD, and IS risks [174]	Mixed: No association [175] Decreased in an APOE ε4 allele-dependent fashion. [176]
rs708272 (+ 279G > A) TaqIB	Decreased [132]	Mixed: Increased MI risk [131] Reduced MI [177] and coronary risks [132] No association with MI [130] and CVR [129]	Mixed: No association [137, 178] Increased in the Asian populations with APOE4 + [179]
rs1532624 (G > T)	Decreased [180]	Mixed: No association with CVR [129] Decreased CAD [171]	Unknown
rs7499892 (C > T)	Unknown, but decreased HDL [181]	Increased CVR [129]	No association [172]
rs5882 (A > G) I405V	Decreased [132]	Mixed: Increased risk of CAD [182] Decreased risk of CHD [183]	Mixed: No association [172, 175, 179] Increased [137, 143] Decreased [142]

*MI* myocardial infarction, *IHD* ischemic heart disease, *ICVD* ischemic cerebrovascular disease, *IS* ischemic stroke, *CVR* cardiovascular risk, *CAD* coronary artery disease, *CHD* coronary heart disease

#### CETP polymorphisms in neurodegeneration and cognitive decline

Several genetic studies support an association between certain *CETP* polymorphisms with resilience to memory decline [135–137]. The Cache County study found that the V allele of the rs5882 (I405V) *CETP* polymorphism was associated with reduced cognitive decline determined by the Modified Mini-Mental State Examination (3MS) in 4486 subjects who were followed longitudinally for 12 years [136]. A protective effect of the V allele of the rs5882 polymorphism in preserving cognitive function measured with the Ruff Figural Fluency Test was also reported in a population-based cohort sample of 4135 individuals in a study in the Netherlands [138]. An Alzheimer Disease Neuroimaging Initiative (ADNI) study investigating *CETP* polymorphisms rs5882 (I405V) and (rs1800775) C-629A in 188 controls and 318 AD or mild cognitive impairment (MCI) patients reported that, in *APOE4* carriers, the *CETP* V and A alleles, both of which decrease CETP activity and increase HDL, were associated with greater cortical thickness at baseline and less atrophy over 12 months in the medial temporal lobe. By contrast, for non-*APOE4* carriers, the I allele, which increases CETP and decreases HDL, was associated with greater baseline thickness and lower dementia risk, suggesting that *APOE* genotype may modify the impact of *CETP* polymorphisms on neurodegeneration and cognitive decline [139]. Diffusion tensor imaging of 403 young adults revealed that the G allele dosage of the rs5882 (A > G) polymorphism was associated with higher fractional anisotropy and lower radial and mean diffusivity, suggesting optimal white matter structural integrity. However, a follow-up analysis of 78 older individuals from the ADNI cohort found an opposite direction of the rs5882 and white matter integrity association, suggesting age-dependent effects on brain [140]. In the Einstein Aging Study, valine homozygosity at the I405V locus was reported to be associated with less incident dementia and slower memory decline determined by the Mini-Mental State Examination in a prospective cohort sample of 608 community-dwelling participants [141]. Another paper from this study showed that *APOE4* significantly interacted with *CETP* I405V, where the V allele buffered the effects of *APOE4* memory decline in a sample of 909 community-dwelling adults [142].

However, other genetic studies suggest a more complex scenario. A 2014 meta-analysis of 9 case control studies including 2172 AD patients and 8017 healthy controls suggested a modest detrimental effect of the rs5882 (I405V) polymorphism in increasing AD risk specifically in Caucasians [137]. The Rush Memory and Aging Project and the Religious Order Study reported that the *CETP* rs5882 polymorphism was associated with increased AD risk in over 1300 participants of European ancestry [143]. A study of 544 AD cases and 5405 controls from the Rotterdam study similarly suggested increased AD risk in VV homozygotes specifically in *APOE4* noncarriers [144]. It is possible that the discrepancies among the genetic studies may be explained by interactions among *CETP* and *APOE* genetic variation, aging, cardiovascular risk factors, and the presence of multiple neuropathological subtypes of AD. Indeed, a 2020 study using Mendelian randomization of the genetic determinants of blood lipids and cerebral small vessel disease showed that a genetic predisposition to higher HDL-C levels, including *CETP* variants, was associated with reduced risk of small vessel stroke and white matter hyperintensities that remained after adjustment for LDL-C and triglycerides [145].

#### Lack of functional CETP expression in mice

Due to a deletion that leads to a nonsense mutation in exon 11 of the rodent *CETP* gene, both mice and rats have no active CETP protein and thus are functionally equivalent to CETP knockouts. Lack of CETP activity is why mice and rats have naturally high HDL and low LDL levels that leads to an inherent resilience to atherosclerosis and, importantly, potential resistance to VCID. Introducing the *CETP* gene into mice increases LDL-C levels and decreases HDL-C levels, and is pro-atherogenic in mice fed an atherogenic diet [146], in apoE knock-out mice, [147] in LDL receptor knock-out mice, [147] in APOE*3-Leiden mice [148] and in hypertensive rats [149]. A recent study of commercially available CETP transgenic (Tg) mice (Jax strain 003904) demonstrated transcriptional changes in the liver including reduced Hmgcr, Ldlr, and Lrp1 levels and increased ABCA7 and Trem2, albeit the Trem2 mRNA levels were not associated with increased Trem2 protein levels as measured by Western blot [150]. This group also reported that CETP was expressed in the cortex of CETP transgenic mice and increased Il-1β levels. Upon induction of CETP activity through a high-cholesterol diet, CETP Tg mice displayed a 22% increase in hippocampal cholesterol levels that was reported to be due not by increased cholesteryl synthesis in astrocytes but rather to decreased cholesterol excretion through the BBB. Decreased cholesterol efflux was revealed by a significant reduction in 24S-hydroxycholesterol, a cholesterol metabolite that can freely diffuse over the BBB, in the brains of CETP transgenic mice [150]. Transcriptional profiling of astrocytes of these mice also revealed high upregulation of complement factor C1Q subunit genes which was associated with increased C1Q protein levels throughout the hippocampus. As C1Q has been associated with neuronal cholesterol efflux [151], increased C1Q protein expression may be a compensatory mechanism to excrete excess cholesterol. C1Q is also a major effector of the peripheral immune response and mediates synapse elimination in the developing brain. Importantly, C1Q is increased in the CNS during ischemia–reperfusion injury, in AD, and during normal aging [152–155], where it is hypothesized to contribute to disrupted hippocampal circuitry [155] and to astrocyte-mediated synapse loss [156]. More work is needed to validate these findings, as a recent systematic review and meta-analysis of 86 studies investigating the complement cascade in AD did not find evidence for consistently altered CSF C1Q levels in AD patients [157], potentially reflecting mixed vascular and parenchymal pathology in the studies reviewed.

### CETP as a therapeutic target

Several CETP inhibitors have been developed and evaluated in clinical trials for cardiovascular outcomes. Torcetrapib inhibited the development of atherosclerosis in rabbits and, in early-phase studies in humans, increased HDL-C by 60 to 100% and lowered LDL-C by up to 20% [158]. However, torcetrapib was associated with increased risk of death and cardiac events due to off-target effects that increased aldosterone, cortisol and endothelin-I, changed serum electrolytes, and increased blood pressure. Since then, other CETP inhibitors have been required to undergo assessments to exclude off-target toxicity and include dedicated ambulatory blood pressure monitoring studies, with dalcetrapib, evacetrapib, anacetrapib and obicetrapib showing no clinically relevant effects on blood pressure or mineralocorticoid levels [159–161]. Although dalcetrapib raised HDL-C levels by approximately 30% in phase 2 studies, lack of significant LDL-C lowering led to termination of clinical trials due to futility [162]. Evacetrapib trials were also halted due to futility, however, this could be due to insufficient duration to detect a meaningful reduction in major coronary events [163]. Anacetrapib was found to have subtle LDL-C lowering effects and significantly reduced major coronary events (i.e., coronary death, myocardial infarction, or coronary revascularization) over 6.4 years of follow up, where continued efficacy results from its accumulation in adipose tissue leading to a long half-life [164, 165]. Torcetrapib, anacetrapib, dalcetrapib and evacetrapib are all lipophilic compounds that block transfer of cholesteryl esters by interfering with the connection between the N- and C-terminal pockets in the CETP protein. Evacetrapib has been reported to enter mouse brain tissue [166], although it is not clear at this time whether CNS entry is required for CETP-inhibitors to potentially affect AD-relevant pathways.

Despite the failed trials listed above, one CETP inhibitor, obicetrapib, is advancing through clinical trials mainly for cardiovascular outcomes (NCT05972278, NCT05142722, NCT05425745, NCT4753606, NCT04770389, NCCT05421078, NCT06005597, NCCT05202509, NCT05266586), with one proof-of-concept open label phase 2a trial in early AD patients to evaluate plasma and CSF lipoprotein changes (NCT05161715). Unlike the other CETP inhibitors, obicetrapib is significantly less lipophilic and shows no clinically significant off-target effects on vital signs, blood pressure, and aldosterone, sodium, potassium or bicarbonate concentrations [161]. Obicetrapib also exerts the most potent effects on LDL-C and HDL-C among the CETP inhibitors tested to date with very favourable changes in lipid profile. Specifically, obicetrapib at a 5 mg dose reduced CETP activity at steady state by 91%, reduced LDL-C by 45.3%, reduced apoB by 33.6%, increased HDL-C after 12 weeks by 157.1%, and increased apoA-I by 57.5% [167]. These attributes make obicetrapib an attractive candidate to evaluate in the context of VCID, as it leads to favourable changes in *both* LDL-C and HDL-C levels. LDL-C reduction is required for lowered cardiovascular risk, which would be expected to reduce LDL-mediated effects on AD pathological changes, and the elevated HDL-C is of interest with respect to its potential beneficial roles in endothelial physiology and amyloid clearance.

### Knowledge gaps and considerations for future directions

#### Improving mouse models for VCID studies 

High levels of vasoprotective HDL and low levels of LDL renders mice highly resilient to vascular disorders, and VCID studies in mice need to be interpreted in this context. One approach to improve the relevance of mouse models for VCID studies, especially studies that aim to understand the contribution of peripheral lipoproteins on ADRD, is to reconstitute functional CETP activity to generate a more human-like peripheral lipoprotein profile in mice. This would be expected to exacerbate CAA and BBB dysfunction. One strain of CETP Tg mice that express a human CETP minigene containing both 5’ and 3’ regulatory elements responsive to a high fat high cholesterol diet is commercially available from Jackson Laboratories (Jax strain 003904) and could enable fundamental research studies to investigate several unanswered questions, such as the effect of CETP on BBB and brain expression profiles, inflammatory tone, cerebrovascular physiology, amyloid deposition, amyloid clearance, and tau pathology. Reconstitution of CETP activity in mice will also be essential to address whether CETP inhibition is effective against CAA, parenchymal amyloid, or both, whether CETP inhibition can remove pre-existing vascular or parenchymal amyloid deposits, whether apoE isoform modifies CETP inhibition efficacy, whether CETP inhibition can reduce apoE-mediated tau neurodegeneration, and whether CETP inhibitors need CNS entry to affect vascular, amyloid, tau, and inflammatory pathways relevant to AD.

#### Improving in vitro models for VCID studies

Although several advanced in vitro models such as vascularized human brain organoids and human BBB models have been developed and applied in VCID studies [168, 169], a major limitation of these models is the absence of human endothelial cells, either primary or iPSC derived, which recapitulate the transcriptomic profiles of human cerebrovascular endothelial cells, including vascular zonation considerations. For multicellular models, much needs to be learned about how transcriptomic profiles of the input cells may change under lengthy co-culture conditions.

#### Understanding whether peripheral lipoproteins modify anti-amyloid safety and efficacy

Given LDL’s causal role in reducing cardiovascular disease risk and association with AD neuropathology, it is of interest to determine whether LDL-C-lowering therapies may also enable existing amyloid deposits to be more efficiently and safely extracted from the brain, and if so, what mechanisms are involved. It will be important to consider the class of LDL-lowering agents, as statins, PSCK9 inhibitors, cholesterol absorption inhibitors (ezetimibe), bile acid sequestrants, ATP citrate lyase (ACL) inhibitors (bempedoic acid), small interfering RNA (siRNA) therapies (Inclisiran) and CETP inhibitors use different mechanisms to lower LDL-C and have different effects on the LDL:HDL ratio that could be important for VCID. Secondary analyses of residual plasma specimens from anti-amyloid immunotherapy trials could reveal whether the LDL:HDL ratio or HDL composition may modify ARIA risk. Similarly, secondary analysis of residual plasma specimens from CETP inhibitor trials could reveal whether AD-relevant biomarkers such as Aβ42:Aβ40, p-tau-181, p-tau-217, neurofilament light, and glial fibrillary acidic protein are altered after exposure to CETP inhibition and whether the degree of biomarker change correlates with the extent of LDL lowering and/or HDL elevation.

## Conclusions

As identification of factors that modify ARIA risk is a major priority, further investigation of peripheral lipoproteins and CETP activity can be considered as attractive candidates. LDL-C, a known cause of cardiovascular disease, is associated with AD pathology and could contribute to cerebrovascular dysfunction in both amyloid-dependent and amyloid-independent ways. How HDL composition affects its known vasoprotective functions is primarily studies in the context of atherosclerosis, and the pre-clinical and in vitro data on the associations of HDL with CAA and Aβ clearance provides a strong rationale for additional studies. There are, however, important challenges to overcome. Many clinical ADRD studies exclude those with vascular disease, and those that do not will have a high proportion of participants with mixed pathology. Better CAA diagnostic and staging tools are needed. Studies in mice need to be interpreted in the context of inherent vascular resilience due to low LDL and high HDL levels. Incorporation of functional CETP into AD animal model studies will improve their relevance to human disease and enable the mechanisms by which lipoproteins and CETP may affect VCID to be understood. An improved understanding of human cerebrovascular endothelial cell physiology in response to lipoprotein exposure will also improve the relevance of human-based in vitro model systems for VCID research.

## Data Availability

Not applicable.

## Abbreviations

Aβ: Amyloid beta
AD: Alzheimer’s Disease
ADNI: Alzheimer’s Disease Neuroimaging Initiative
ApoAI: Apolipoprotein A1
ApoB: Apolipoprotein B
ApoE: Apolipoprotein E
ARIA: Amyloid related imaging abnormalities
BBB: Blood brain barrier
CAA: Cerebral amyloid angiopathy
CETP: Cholesteryl ester transfer protein
CNS: Central nervous system
HDL: High density lipoprotein
HDL-C: High density lipoprotein cholesterol
LDL: Low density lipoprotein
LDL-C: Low density lipoprotein cholesterol
LDLR: Low density lipoprotein receptor
LRP1: Low density lipoprotein receptor related protein 1
PCSK9: Proprotein convertase subtilisin/kexin type 9
SR-B1: Scavenger receptor B1
VCID: Vascular contributions to cognitive impairment and dementia
